# Genetic diversity and population structure analysis of mango (*Mangifera indica* L.) germplasm using microsatellite markers

**DOI:** 10.1186/s12870-026-08892-1

**Published:** 2026-05-08

**Authors:** Madiha Tahir, Shazia Iram, Daniel Potter, Rashid Mehmood Rana, András Székács

**Affiliations:** 1 Department of Environmental Sciences, Fatima Jinnah Women University, The Mall, Rawalpindi, 46000 Pakistan; 2 Department of Plant Sciences, University of California, Davis, One Shields Ave, Davis, CA 95616 USA; 3 Department of Plant Breeding and Genetics, Pir Mehr Ali Shah-Arid Agriculture University Rawalpindi, Shamsabad, Muree Road, Rawalpindi, 46000 Pakistan; 4 Agro-Environmental Research Centre, Institute of Environmental Sciences, Hungarian University of Agriculture and Life Sciences, Páter Károly u. 1, Gödöllő, H-2100 Hungary

**Keywords:** Genetic diversity, Nanophotometer, SSR markers, Principal Coordinate Analysis (PCoA), Analysis of Molecular Variance (AMOVA), Marker-Assisted Selection, Phylogenetics

## Abstract

**Background:**

Mango (*Mangifera indica L.*), known as the “king of fruits,” is a major tropical fruit belonging to the Anacardiaceae family. Cultivated for over 4,000 years, it is native to southern Asia, eastern India, and Burma. The mango family includes 73 genera and 850 species, mostly found in tropical regions. In Pakistan, mango is the second most cultivated fruit after citrus, primarily grown in Punjab and Sindh. Key varieties include Sindhri and Chaunsa, with 7–10% of the crop exported annually, generating about $20 million.

**Results:**

This study aims to assess the genetic diversity of Pakistan’s mango germplasm using simple sequence repeat (SSR) markers for effective crop breeding. Mango germplasm samples were collected from Multan Mango Research Institute. Both local and exotic varieties were included. Genomic DNA was extracted from mango leaves using a Plant Genomic DNA Kit, and its quality was assessed via spectrophotometry. The study aimed to evaluate genetic relatedness among 38 mango cultivars of Pakistan and other countries using SSR primers. Twelve labeled primers were tested for their amplification efficiency through PCR, using labeled forward and unlabeled reverse primers. This study on mango germplasm from Pakistan, using SSR markers, revealed significant genetic diversity among 38 native and exotic cultivars. All primer sets produced clear bands, confirming well-optimized PCR conditions. SSR analysis showed high allelic variation, useful for improving traits like disease resistance, fruit quality, and yield. Principal Coordinate Analysis (PCoA) and cluster analysis indicated both separation and overlap between local and exotic varieties, with some clustering based on geographic origin. Most genetic variation was found within cultivars, highlighting a strong genetic base in Pakistani mango germplasm.

**Conclusions:**

The study confirmed close genetic links between Pakistani mangoes and those from the Philippines, Indonesia, and Thailand, offering valuable insight for future breeding programs. This study demonstrated the potential of molecular marker techniques to enhance mango breeding by selecting high-yield, disease-resistant, and climate-tolerant varieties. Emphasizing the importance of genetic diversity, it addressed future challenges like climate change and disease outbreaks. Based on the findings, a strategic plan was proposed to strengthen germplasm collection and develop climate-smart mango varieties suited for modern production systems. Overall, the research offers valuable insights into mango genetics and presents an effective approach to boost mango production in Pakistan and globally.

## Background

Mango (*Mangifera indica* L.), often referred to as the “king of fruits”, is a member of the Anacardiaceae family and ranks among the most popular tropical fruits worldwide [1, 2]. It holds a leading position in terms of production and economic value among tropical fruits. There are two primary gene centers of mango domestication: one in South Asia (notably India) and another in South-East Asia. These centers have significantly influenced the genetic diversity of mango cultivars, both reaching global geographic distribution [3–5]. Mango varieties from South Asia have been introduced to various regions across the globe, including Asia (India, Pakistan, Sri Lanka, Burma, Iran, China, Japan, and Israel), Africa (Guinea, Egypt), the Americas (the United States, Cuba, the Caribbeans), and to Australia [6–25]. Additionally, hybrids between varieties from these two gene centers are also cultivated [26].

Mango is one of the principal fruit crops cultivated in Pakistan, with the provinces of Punjab and Sindh serving as primary production regions in the country [27]. Mangoes are significant in Pakistan as fresh fruit for the domestic market as well as export, while Sindhri, Chaunsa, and Anwar Ratol brands are prestigious due to their acceptable quality in terms of taste, aroma, and texture [28]. Mango trade is an important income earner and source of employment for millions of people in the country. Nonetheless, the mango industry has been subjected to various impending issues, such as climate change, pest problems and diseases, as well as the narrow genetic base within given germplasms [27, 29, 30]. In Pakistan, repositories in Multan and Sindh centers contain diverse mango germplasms, indigenous and exotic, both important for genetic conservation and improvement. The *ex situ* conservation of mango germplasm is another significant aspect of the unpredictability of mango production. Mango germplasm banks are important in the conservation of mango genetic resources, such that high-yielding varieties can be regenerated for use in upcoming breeding processes. Reciprocal exchanges and conservation of germplasm, and utilizing advanced modern tools of biotechnology can increase mango production and resilience to climate and market changes.

Genetic variation is one of the important prerequisites for constant yield and long-term productivity in the agricultural business. Mango cultivars are less genetically diverse, which greatly restricts the development of improved varieties that will be resistant to pests, diseases, as well as biotic stresses [31, 32]. In addition, single nucleotide polymorphic markers correlating with fruit quality and yield have been identified and applied in mango breeding [23, 33–36]. It is seen that over-dependence on a few cultivars, along with a few elements like inbreeding, has resulted in low genetic diversification amongst the mango cultivars in many producer countries, including Pakistan. This is particularly dangerous, following its impacts on the possibility of profitable mango farming in the future. Furthermore, climate and changing conditions for agriculture in the world, it is even more crucial to know and conserve genetic potential, which will help crops overcome new difficulties [13].

Structural relatedness or simple sequence repeat (SSR) markers, also known as microsatellite markers, prove more effective for the estimation of genetic divergence since the markers are allele-specific and highly polymorphic, providing a high resolution throughout the genome [14, 37, 38]. These markers are broadly distributed throughout the genome, predominantly in noncoding areas e.g., intergenic loci, introns, and untranslated regions, and may contain perfect repeats of a single motif, imperfect sequence varieties with a base pair disruption between repeats, and multiple types of repeat motifs [17]. The application of SSR markers in this study allowed us to obtain a detailed description of the genetic variation within a number of mango cultivars and identified the possibility of conservation and improvement of mango germplasm for the breeding program. Nevertheless, there is still a limitation of genetic diversity; therefore, advanced or new plant breeding techniques that involve the use of molecular biology tools are being developed [5, 39]. Knowledge of genetic information empowers the mango breeding programs to achieve higher aeration and equip the plants with better disease-tolerant types of mango fruits with improved standards of quality and better capability to improve environmental changes [27, 40]. In addition, the availability and use of more exotic germplasm for continued generations of mangoes are advocated for as a way of ensuring that future generations of mangoes yield high productivity and are well adapted to a changing climate and market requirements. The cultivated area and mango production affect prices, but pre- or post-harvest management also has an impact due to its perishable nature and seasonality in arrival [41–43]. Appreciation of the genetic variability and architecture of the mango cultivars is important in efforts to improve the mango varieties, as ongoing challenges persist. The differentiation among the population structure of mangoes could be separated into different gene pools, suggesting the existence of separate ecotypes of genetic diversity. Researchers discovered several potential loci and dominant genotypes linked to mango blooming capacity, fruit weight, and volatile chemical production by combining genome-wide association studies with investigations of genotype variation patterns and expression patterns [23].

This study aims to assess the genetic diversity among mango cultivars using molecular methods via SSR markers. The technique of SSR genotyping allows analysis of genetic variability within species using DNA amplification by polymerase chain reaction (PCR) and detection of the DNA product. Therefore, this study did not require DNA sequencing; it aimed to discover quantitative trait loci for the major characteristics that can be targeted in marker-assisted selection to develop superior mango genotypes. By using only SSR markers, the study is useful in directing the improvement of mango production in Pakistan and as other countries through the understanding of the genetic composition of the mango germplasm. The use of SSR markers in Pakistani mango germplasm analysis is promising due to the ability to provide thorough, molecular-level insights on the genetic diversity, conservation, and future development of mango cultivars. By making it simpler to identify, preserve, and improve mango germplasm for both domestic consumption and export, SSR markers support the future growth of Pakistan’s mango industry.

## Materials and methods

### Plant material

In order to guarantee the accuracy and dependability of subsequent genomic analyses, the study’s initial phase comprised the collection of plant samples. The viability of obtaining the necessary mango types was given particular consideration during the sample collecting procedure, taking into account variables including availability, genetic diversity, and regional adaptability. The samples were collected from the Multan Mango Research Institute’s (MMRI), Mango Germplasm Unit in Punjab, Pakistan, to guarantee their purity and validity. With a wide variety of mango cultivars, MMRI is a respectable hub for mango research and conservation. The study had access to precisely identified and well-maintained germplasm by obtaining samples from this institute. Based on their significance in local cultivation, unique genetic characteristics, as well as potential for breeding and enhancement initiatives, 38 following mango cultivars (Table 1) were chosen for the genomic study. These types are essential for comprehending the genomic diversity of mangoes in Pakistan because they reflect a wide range of genetic backgrounds.

**Table 1:** Mango varieties selected for the study

Sample ID	Name of variety	Origin	Sample ID	Name of variety	Origin
M1	Fajri	Sindh, Pakistan	M20	Rupee	Indo-Pak
M2	Falan	Thailand	M21	Chenab Gold	Sindh, Pakistan
M3	SB Chaunsa	Indo-Pak	M22	Palmer	Australia
M4	Pirie	Hawaii	M23	Crimson Blush	Australia
M5	Australian Common	Australia	M24	Late Sindhri	Sindh, Pakistan
M6	Alishan	Punjab, Pakistan	M25	Kala Chaunsa	Punjab, Pakistan
M7	Safeed Chaunsa	Punjab, Pakistan	M26	Indo Chinese late	Indonesia
M8	Mosami Sindhri	Sindh, Pakistan	M27	Carabao lemon	Philippine
M9	Dusheri	Indo–Pak	M28	Jakarta	Indonesia
M10	Banana Long	Thailand	M29	Rohan	Punjab, Pakistan
M11	Samar Behisht	Punjab, Pakistan	M30	Kensington Pride	Australia
M12	Ratol No 12	Punjab, Pakistan	M31	Chaunsa	Punjab, Pakistan
M13	Keow Savoy	Austrilia	M32	Tong Dum	Thailand
M14	Langra	Indo-Pak	M33	Olur	Mexico
M15	Carabao Timiteo	Phillipine	M34	Sindhri	Sindh, Pakistan
M16	Maha	Thailand	M35	Malda	Indo-Pak
M17	Bullock’s Heart	Thailand	M36	Hasan	Punjab, Pakistan
M18	Brown Seedling	Thailand	M37	Azeem Chaunsa	Punjab, Pakistan
M19	Anwar Ratole	Indo-Pak	M38	Elephant Tusk	Thailand

### Genomic DNA extraction, quantification and multiplex PCR amplification

Each accession’s leaf tissues were used to obtain genomic DNA. After using liquid nitrogen to grind the leaves into a fine powder, they were incubated in an extraction buffer at 65 °C for ten minutes. Following cooling to 20 °C, the mixture was emulsified by adding an equivalent volume of chloroform/isoamyl alcohol (24:1, v/v). After that, the tubes were centrifuged for 20 min at 10,000 × g. Cold isopropanol was used to precipitate the DNA from the resultant supernatant. The NanoDrop 2000 C spectrophotometer (Thermo Scientific, USA) was used to measure the extracted DNA’s quality and concentration [13]. For the examination of DNA fragments, twelve primer pairs (Table 2) were created. Initially, DNA from various mango accessions was used to screen these primers for polymorphism and amplification efficiency. Multiplex PCR was used to amplify the DNA after fluorescently labeled primers were chosen [9].

**Table 2:** Selected Forward (F) and Reverse (R) primers for analysis of mango accessions [44–46]

Primer	Sequence (5’ to 3’)
M01:F	GGATGCACAACAACAAGAC
M01:R	TCAGCAAGCAATCCCTTCTT
M05:F	CTCTCCCTCACTTGCTCCAC
M05:R	AGACCACCGACAACGAAAAC
M07:F	GCCACTCAGCTAAATAGCCTCT
M07:R	TGCAGTCGGTAAAGTGATGG
M09:F	GTTGTGACCGAGGCCTTAAA
M09:R	CTTTGACATCGCTGATCTGG
M10:F	CGATTCAAGACGGAAAGGAA
M10:R	TTCAAGCACAGACGACCAAC
M11:F	CAGTGAAACCACCAGGTCAA
M11:R	TGGCCAGCTGATACCTTCTT
M14:F	CCGAAACAACTCTTCCTCCA
M14:R	TGCTCTCTGGCCTCTTCTTC
M22:F	TGGCCGAACTAGCAAACTCT
M22:R	CCCCATTTCGAGAAAATTCC
M24:F	GCTCAACGAACCCAACTGAT
M24:R	TCCAGCATTCAATGAAGAAGTT
M30:F	AGCTATCGCCACAGCAAATC
M30:R	GTCTTCTTCTGGCTGCCAAC
M33:F	GAAGCACTTGTCTCCCTTGC
M33:R	CCTCACACTCCTCCACCTGT
M36:F	TCTATAAGTGCCCCCTCACG
M36:R	ACTGCCACCGTGGAAAGTAG

All PCR reagents were kept on ice during the preparation process to preserve the integrity of the samples and reagents as well as to avoid the thermal degradation of the proteins and enzymes being studied [47]. Primer dilutions were made in multiplex reactions before fragment analysis on the genetic analyzer.

### Data analysis and determination of genetic diversity

The selection of SSR primers was carried out using already generated mango SSR from databases or available resources. Primers were chosen based on their high PIC value, coverage across various chromosomes, and evident amplification of the DNA from mango leaves. SSR primers are used to amplify the DNA of mango cultivars. Different fragment sizes of alleles are produced by each SSR locus. An internal size standard is used to score allele sizes (in base pairs). The alleles of each cultivar at each locus are listed in a genotyping table that is created from the data. Two alleles per locus were scored co-dominantly (homozygous = twice the same size, heterozygous = twice different sizes). Major allele frequency (MAF), observed heterozygosity (Ho), expected heterozygosity (He), polymorphic information content (PIC), population differentiation, number of alleles per locus (Na), and AMOVA to divide variation between and within cultivars were the genetic diversity indices.

Basic genetic statistics such as allele number and observed or predicted heterozygosities for each locus and species were calculated using GenAlEx software (Genetic Analysis in Excel v.6.5) [48–50]. These metrics provided insights into the levels of genetic variation present across the mango genotypes. Principal Coordinate Analysis (PCoA), based on microsatellite marker data, was also carried out using GenAlEx to visualize the genetic relationships among accessions. Furthermore, Bayesian clustering analysis was performed using the software STRUCTURE (version 2.3.4) to infer population structure and determine the optimal number of genetic clusters among the sampled genotypes [51, 52]. For further investigation genetic variation, an AMOVA were conducted to partition diversity within and among the 38 different mango genotypes. F-statistics were applied to quantify the degree of genetic differentiation between populations [53, 54].

## Results

In order to evaluate the genetic diversity among 38 mango (*Mangifera indica*) cultivars from a wide geographic range, including Pakistan, the Philippines, Mexico, Florida (USA), Thailand, Australia, and Indonesia, a set of twelve fluorescently labeled SSR primers was chosen. These primers were carefully selected based on a number of important factors, including their capacity to produce distinct and clear amplicons during gel electrophoresis, high amplification efficiency, ideal fragment length, adequate guanine-cytosine (GC) content, and suitable annealing temperatures. The efficiency of the primers in generating repeatable and comprehensible molecular data was guaranteed by these characteristics.

These primers’ broad application and dependability in genotyping varied genotypes are demonstrated by the effective targeting of the SSR loci across all mango samples by PCR amplification. The SSR primers were fluorescently tagged with one of three dyes—hexachlorofluorescein (HEX), carboxyfluorescein (FAM), or N-(1-naphthyl) ethylenediamine (NED)—to enable automated detection and size via capillary electrophoresis and to assist high-resolution fragment analysis.

MiIIHR01, MiIIHR05, MiIIHR07, MiIIHR09, MiIIHR10, MiIIHR11, MiIIHR12, MiIIHR14, MiIIHR22, MiIIHR30, MiIIHR33, and MiIIHR36 were among the primers in the set. Comprehensive genetic diversity studies were based on the polymorphic and scorable alleles that were consistently produced by these forward and reverse primer combinations. The SSR markers were appropriate for evaluating genetic linkages, population structure, and allelic richness among the chosen mango cultivars due to their robust and polymorphic nature (Table 3).

**Table 3:** Characteristics of 12 simple sequence repeat primer pairs for the genetic diversity of the collected germplasm of mango

Primer	Sequence (5’ to 3’)	Repeat motif	No. of alleles
MiIIHR01:F MiIIHR01:R	GGATGCACAACAACAAGCAC TCAGCAAGCAATCCCTTCTT	(GAA)_4_CAG(CAA)_2_(TA)_2_	7
MiIIHR05:F MiIIHR05:R	CTCTCCCTCACTTGCTCCAC AGACCACCGACAACGAAAAC	(CT)_8_C(CT)_2_TTTT(CT)_4_	13
MiIIHR07:F MiIIHR07:R	GCCACTCAGCTAAATAGCCTCT TGCAGTCGGTAAAGTGATGG	(GA)_11_	6
MiIIHR09:F MiIIHR09:R	GTTGTGACCGAGGCCTTAAA CTTTGACATCGCTGATCTGG	(CT)_3_TTGC(CT)_2_GT(CT)_4_ TC(GT)_2_(CT)	5
MiIIHR10:F MiIIHR10:R	CGATTCAAGACGGAAAGGAA TTCAAGCACAGACGACCAAC	(GTT)_26_	7
MiIIHR11:F MiIlHR11:R	CAGTGAAACCACCAGGTCAA TGGCCAGCTGATACCTTCTT	(CT)_2_TT(CTT)_5_	3
MiIIHR12:F MiIIHR12:R	GCCCCATCAATACGATTGTC ATTTCCCACCATTGTCGTTG	(GA)_11_	9
MilIHR14:F MiIIHR14:R	CCGAAACAACTCTTCCTCCA TGCTCTCTGGCCTCTTCTTC	(GAA)_3_(AG)_2_A(AAG)_3_AG(GAA)_2_GGA(GAAA)_2_AA(GAA)_3_	6
MiIIHR22:F MiIIHR22:R	TGGCCGAACTAGCAAACTCT CCCCATTTCGAGAAAATTCC	(GTCTC)_2_(TGTCTC)_3_T (CTC)_2_	7
MiIIHR30:F MiIIHR30:R	AGCTATCGCCACAGCAAATC GTCTTCTTCTGGCTGCCAAC	(CT)_13_	11
MiIIHR33:F MiIIHR33:R	GAAGCACTTGTCTCCCTTGC CCTCACACTCCTCCACCTGT	(GA)_12_	4
MiIIHR36:F MiIIHR36:R	TCTATAAGTGCCCCCTCACG ACTGCCACCGTGGAAAGTAG	(TC)_17_	12

### Genetic diversity analysis

**Fig. 1:**
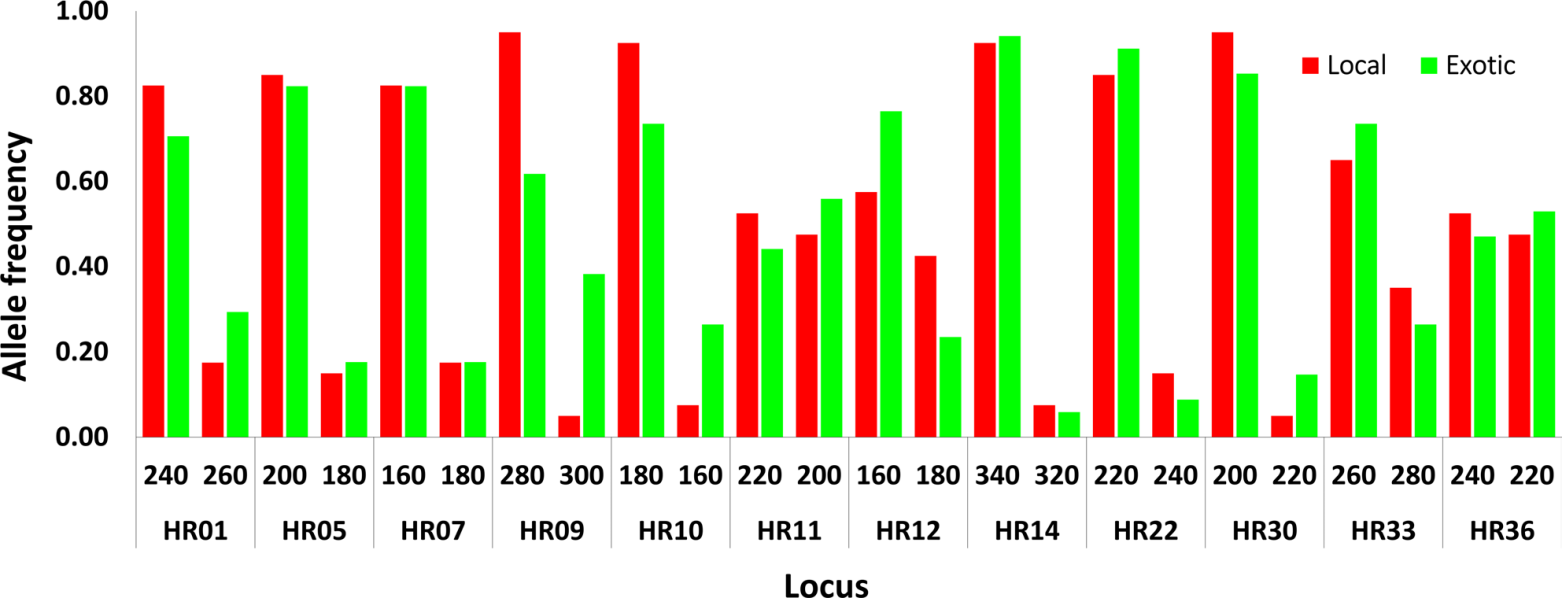
Allele frequency in both local (red) and exotic (green) cultivars of mango germplasm

Local and foreign mango cultivars’ allele frequencies at different SSR loci are contrasted in this bar graph. The y-axis displays allele frequency values between 0 and 1, while the x-axis displays the various SSR loci (HR01 to HR36) and their corresponding allele sizes (in base pairs). There are two color-coded categories: yellow bars indicate local mango cultivars, while grey bars indicate foreign mango cultivars. Each location in allele frequency variation highlights genetic divergence by displaying variations in allele frequencies between local and exotic groups. HR09 (allele 300) and HR22 (allele 240) are private or dominant alleles that are almost exclusively found in local cultivars and are extremely seldom seen in exotic ones. On the other hand, HR12 (allele 160) and HR14 (allele 340) exhibit low frequencies in local cultivars and high frequencies in alien cultivars, indicating allele specialization or unique alleles in particular populations. Locations shared with frequency variations in loci, such as HR01, HR07, and HR30, reveal alleles that are present in both populations but have notably different frequencies, suggesting either geographical adaptation or possible selection pressure. In diversity and polymorphism, multiple alleles with moderate frequencies are found in loci such as HR33 and HR36, indicating significant polymorphism, which is useful for diversity research. Based on SSR marker allele frequencies, this graphic shows how native and exotic mango genotypes differ genetically. It implies that there is significant genetic variability at some loci and that some alleles are unique or highly enriched in either local or exotic groups, which makes them valuable for investigations of population structure and diversity (Fig. 1).

### Principal Coordinate Analysis (PCoA)

**Fig. 2:**
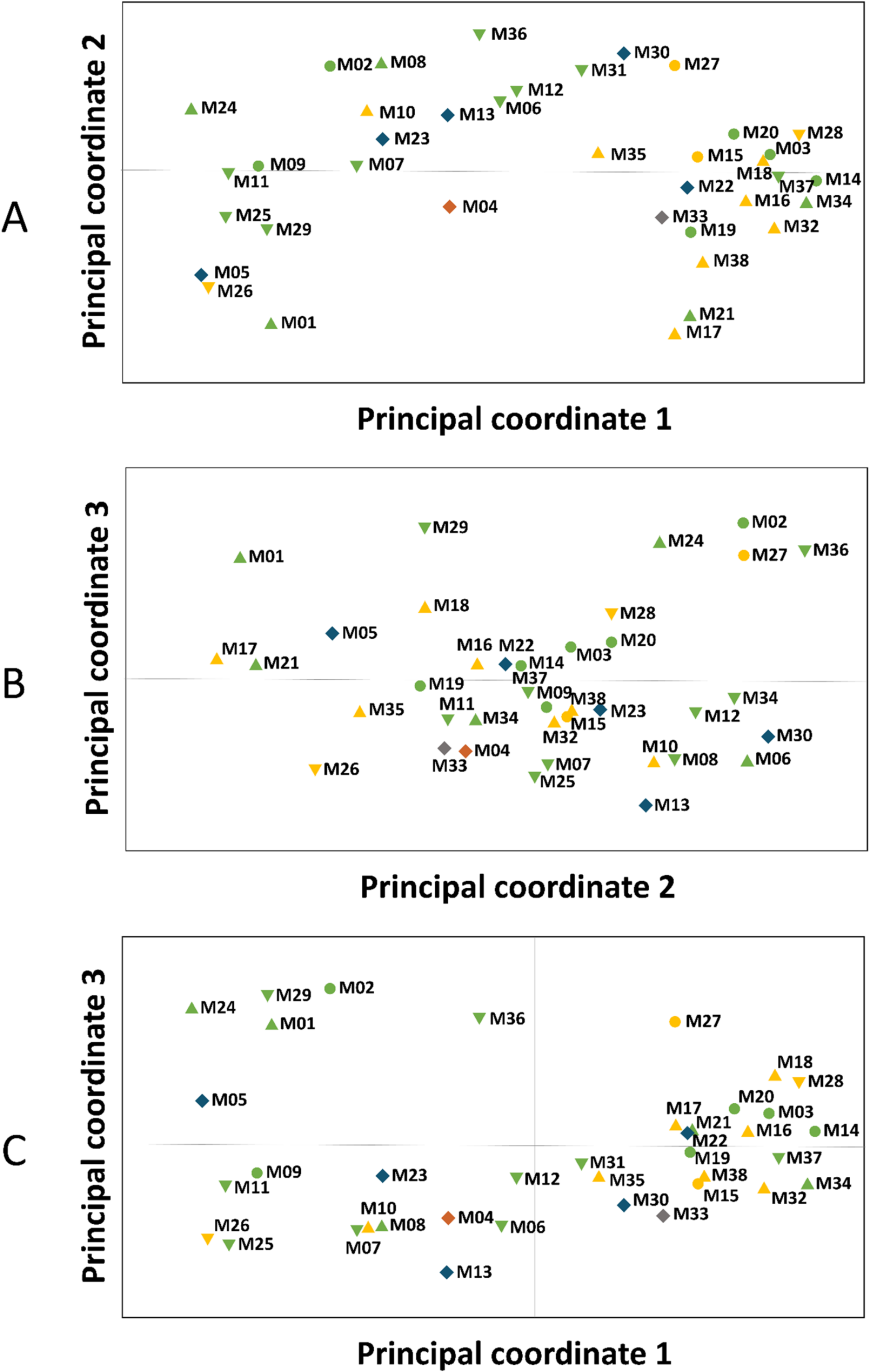
Principal coordinate analysis of the genetic relationships among 38 mango cultivars based on simple sequence repeat data. Origin of mango cultivars: Indo–Pak (), Pakistan, Sindh province (), Pakistan, Punjab province (), Thailand (), Indonesia (), Philippines (), Australia (), Hawaii (), Mexico (). Visualization of pairwise correlations among principal coordinates. **A**: Scatter plot of principal coordinate 1 vs. principal coordinate 2. **B**: Scatter plot of principal coordinate 2 vs. principal coordinate 3. **C**: Scatter plot of principal coordinate 1 vs. principal coordinate 3

M03, M15, M16, M18, M20, M28, and M34 are among the groups that exhibit tight clustering, indicating that they are genetically similar possibly from the same variety group or geographic origin. Greater genetic divergence is indicated by the isolation of some genotypes, such as M04, M01, and M36, from the others. A considerable degree of genetic variation among the 38 mango cultivars is suggested by the genotype distribution throughout all four quadrants. In particular, when various symbol types are found close to one another, this illustrates admixture or shared ancestry among groups (Fig. 2A). The second and third most significant components of variation among the samples are represented by Principal Coordinates 2 (x-axis) and 3 (y-axis), respectively. Each point’s location indicates the degree of similarity or difference between the samples according to the selected distance metric. In the upper right corner, M24, M27, and M36 are grouped together, which would suggest a group with related traits. The lower right quadrant contains M06, M08, M10, and M30, which may be a separate group. Given how different M01 and M05 are from the majority of samples, it seems likely that they are outliers in this coordinate space (Fig. 2B). Figure 2C indicates that certain groupings (such M17, M21, and M22) cluster closely together, indicating a high degree of similarity. Others (such M05 far left and M27 far right) are isolated, indicating that they deviate significantly from the majority of samples.

By visually mapping genetic variation in scatter plots, the PCoA analysis of SSR data identified genetic links across 38 mango cultivars. The variance was explained by principal coordinates 1, 2, and 3 in the following proportions: 10.50%, 5.49%, and 5.15% (total 21.15%). Plotting revealed that unique cultivars were farther apart and genetically similar cultivars were closer together. There was no discernible geographic origin-based clumping, indicating that genetic organization was not significantly influenced by geography. A partial overlap between native and exotic cultivars, however, suggested common ancestry or gene flow, while some separation between them revealed genetic distinctions.

### Genetic diversity and allelic distribution by region

The analysis of mango cultivars across regions (Table 4) revealed varying levels of genetic diversity, with some areas displaying high variation and unique genetic resources, while others demonstrated lower diversity. High genetic diversity is indicated by a PIC near 1.0. The greatest PIC (0.72) is found in region 3, indicating that markers are very informative in this area. With the lowest (0.60), region 4 has the least amount of variability. Fixed alleles unique to specific regions and prevalent alleles shared across multiple areas were identified (Table 5).

**Table 4:** Genetic diversity metrics among collected mango germplasm by region

Region	No. of cultivars	Average No. of alleles per locus	Average PIC* value	Genetic distance (mean)
Region 1	10	4.2	0.68	0.15
Region 2	8	3.8	0.65	0.20
Region 3	12	4.5	0.72	0.25
Region 4	8	3.7	0.60	0.30

*PIC Polymorphic information content

**Table 5:** Unique and common alleles across collected germplasm of mango by region

Region	No. of unique alleles	No. of common alleles	Total No. of alleles
Region 1	2	3	5
Region 2	3	2	5
Region 3	4	4	8
Region 4	1	3	4

While regions 1 and 2 exhibit modest diversity (5 alleles each), area 3 exhibits the highest genetic richness (most total and unique alleles). The least diverse region is region 4 (4 alleles, 1 unique).

### Analysis of Molecular Variance (AMOVA)

In population genetics, Analysis of Molecular Variance (AMOVA) tables are frequently used to illustrate the distribution of genetic diversity. The AMOVA results revealed that the majority of genetic variation (60%) is found within individuals of the same mango population, indicating substantial diversity within cultivars. Approximately 38% of the variation exists among individuals within populations, reflecting genetic differentiation within populations, while only 2% of the variation is observed among populations, suggesting minimal population structure and substantial gene flow that results in limited genetic differences across regions (Table 6).

**Table 6:** Analysis of molecular variance from simple sequence repeat data of mango cultivars

Source	Degrees of freedom	Sum of squares (SS)	Mean sum of squares (MS)	Variance of the mean estimated	Percentage
Among populations	7	57.257	8.180	0.081	2%
Among individuals	30	223.229	7.441	2.076	38%
Within individuals	38	125.000	3.289	3.289	60%
Total	75	405.487	18.910	5.446	100%

* Fixation indices by F-statistics: F_IS_ = 0.387, F_ST_ = 0.015, F_IT_ = 0.396, F_ST_(max) = 0.106

### Analysis of population structure and admixture analysis

**Fig. 3:**
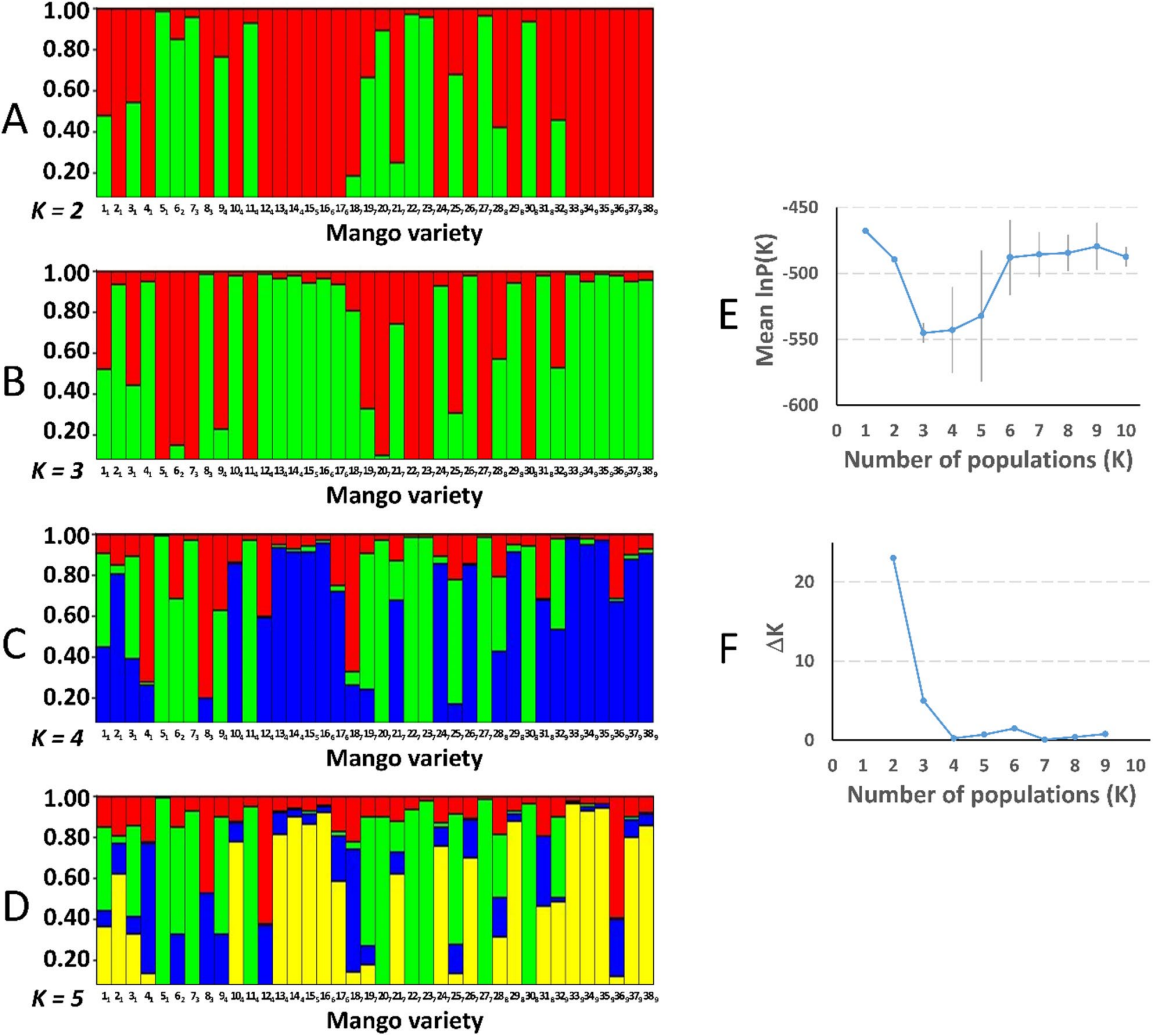
Population structure analysis of 38 mango genotypes based on the number of fitted populations (K) from 2 to 5 and corresponding ΔK values. Two populations (K = 2), local (red) and exotic (green) (**A**); three populations (K = 3) (**B**); four populations (K = 4) (**C**); five populations (K = 5) (**D**). Graphical presentation of the estimation of posterior probability LnP(K) (**E**) and ΔK (**F**)

Figure 3 displays the population structure of the 38 mango genotypes studied. Population structures were modeled with numbers of fitted populations of 2 (Fig. 3A), 3 (Fig. 3B), 4 (Fig. 3C), and 5 (Fig. 3D) considered. The natural log posterior probability of the data (the LnP(K) score) as a function of the number of fitted populations (K) and the corresponding ΔK values are also depicted (Fig. 3E and F, respectively). A ΔK peak value occurred when K was 2 (Fig. 3F). Based on the greatest probability value concept and the K value determination technique it was determined that the optimal K value was 2, which suggested that two groups of 38 individuals are genetically diverse from each other. Consequently, 38 mango germplasm resources were categorized into two subpopulations (Fig. 3A). Red color consists of local varieties and the green color consists of exotic varieties. The Bayesian population structure of the mango genotypes yielded ad hoc statistic ΔK value K = 3 and 4.

In the population structure graphs, each vertical bar represents a single mango variety, and its color segments show the proportion of its genome that belongs to each of the clusters of assumed population number (K). Therefore, in the graph with two populations (K = 2), 60% red and 40% green and shows ancestry from both clusters. K = 3 means the model infers three ancestral populations and each bar can have up to three color segments, and showing the proportion of the genome that comes from one of the three clusters, although only red and green are distinctly visible here. K = 4 is another population structure bar plot, showing the genetic structure of mango varieties assuming 4 ancestral genetic clusters. The color segments show the percentage of ancestry from each of the four clusters. K = 5 image represents graphical output commonly used in population genetics to infer population structure using multi-locus genotype data. Each color corresponds to one of the five inferred genetic populations. The relative height of each color within a bar indicates the proportion of the individual’s genome derived from that population. A mango variety with a single solid color bar (e.g., mostly yellow or green) is genetically homogeneous and assigned to one cluster/population. A mango variety with multiple colors in its bar has admixture, meaning it has genetic ancestry from more than one of the five inferred populations. This plot helps identify population structure, hybridization, and genetic diversity among the 38 mango varieties.

**Fig. 4:**
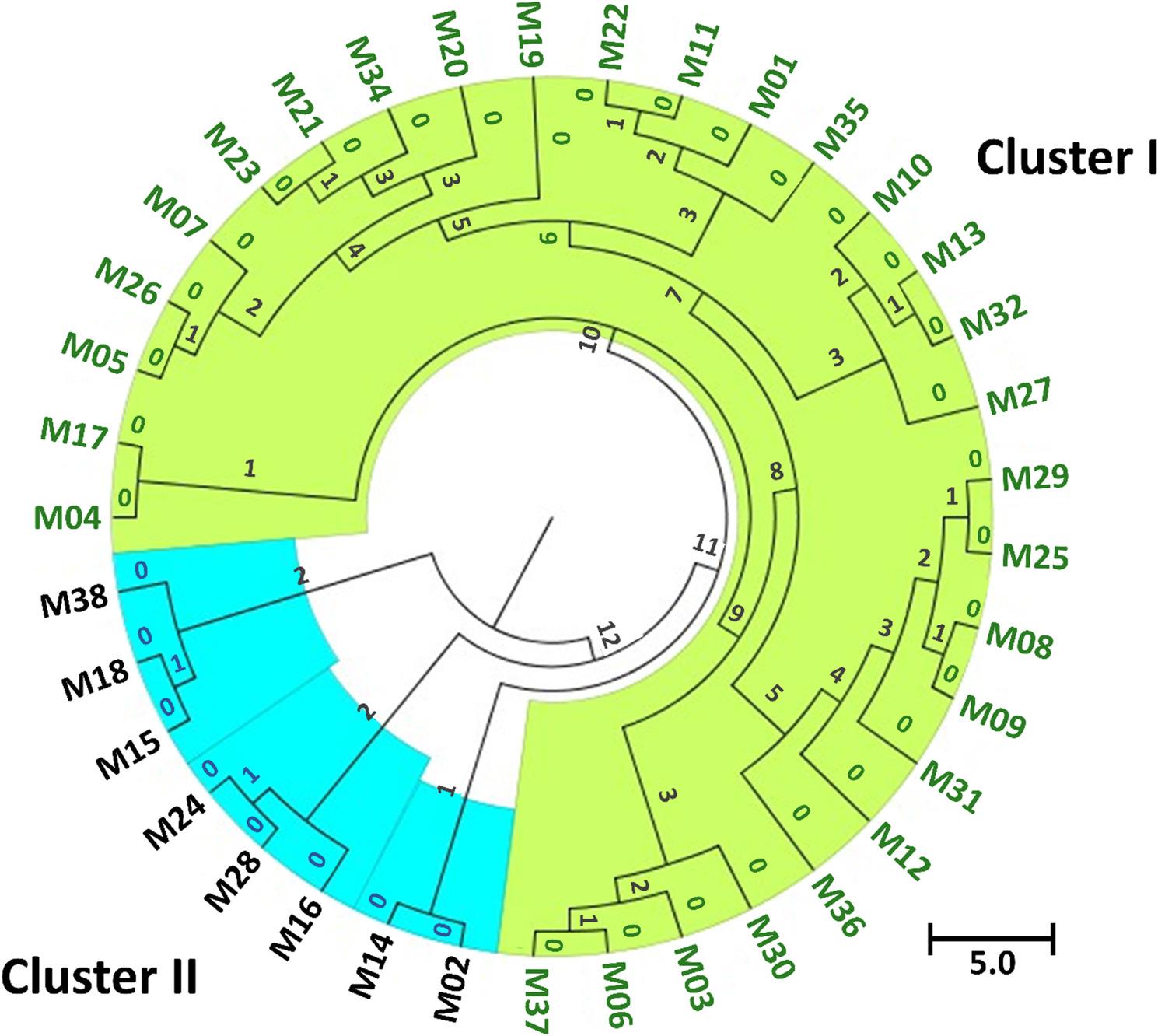
Circular dendogram developed for 38 *Mangifera* cultivars

If the analyses had provided strong support for some number of genetic subgroups, then at that K levels we would expect to see many of the samples showing as just one color. In addition, if the genetic subgroups corresponded to the geographic origins of the samples, then we would expect, for example, that all samples from Thailand would show up as one color and those from Australia as a different color. Since such patterns are not observed at any of the K values tested, it is concluded that there is no evidence for population structure among the individuals sampled, consistent with the results from AMOVA and PCoA. Cluster analysis was performed to sort the mango cultivars into different clusters and each cluster was comprised of individuals of similar genetic profiles. The information obtained in the course of the analysis indicated that some groups of cultivars might originate from different genomic backgrounds or have been exposed to different processes of selection throughout the process of cultivation and, subsequently, breeding. Additionally, the grouping pattern observed in the cluster analysis may be due to the geographic origins of cultivars as the cultivars from the given regions are mostly clustered. The result of the cluster analysis is presented in Fig. 4, which gives us a clear picture of the genetic distance matrix of mango cultivars.

### Phylogenetic analysis of mango species

A phylogenetic analysis was conducted to assess the evolutionary relationships and genetic relatedness between various mango species. Large datasets can be shown more easily because the tree is depicted in a circular shape rather than a straight line. The evolutionary or genetic divergence between mango samples is represented by each branch. These most likely reflect genetic distance measurements or bootstrap values, which show branching confidence.

The analysis revealed two main clusters: Cluster I: Comprising 30 mango varieties, primarily from Australia, Indonesia, Pakistan, Mexico, and the Philippines. Cluster II: Including 8 mango varieties, mainly from Thailand, the Philippines, and Indo-Pak regions. The results indicate that mango varieties from Australia, Mexico, and the United States share a closer genetic relationship with Pakistani mango varieties compared to those from Thailand, Indonesia, and the Philippines. Cluster II demonstrated a closer genetic affinity between the germplasms of Thailand, Indonesia, and the Philippines. This phylogenetic information enhances our understanding of the genetic relationships among mango cultivars and informs breeding strategies by identifying potential sources of genetic diversity.

## Discussion

In this study, emphasis was placed on the use of SSR markers on genetic variations among mango cultivars. Fragment analysis was conducted to amplify twelve SSR markers by multiplex PCR using fluorescently labeled forward primers and unlabeled reverse primers to analyze the diversity of the amplified DNA products across the mango cultivars studied. Multiplex PCR allows for simultaneous detection of multiple DNA targets in a single reaction by labeling primers, frequently with fluorescent dyes. This labeling is essential for differentiating the PCR products produced by various primer sets, which enables researchers to simultaneously detect and measure several DNA sequences. This work allowed assessment of genetic diversity in mango germplasm. The assessment of mango germplasm collection and morphological diversity in Pakistan is considered a prerequisite for the maintenance of genetic variability and for enhancing the ongoing breeding programs to develop better varieties of mango. The germplasm accessions belonging to different parts of Pakistan, such as Punjab, Sindh, KPK and Baluchistan have potential genetic resource banking essential for the future perspective of mango production. The genetic diversity of 13 mango cultivars was evaluated by Bukhari et al. [21] using 28 SSR markers. The mean values of 3.92 (alleles), 0.45 (minor allele frequency), 0.54 (gene diversity), and 0.50 (PIC) were reported. Indicating a variety of ancestry among genotypes, the study emphasized heterozygosity resulting from spontaneous hybridization and farmer-led selection of somatic mutants. SSR markers improved knowledge about the organization of mango germplasm by effectively differentiating closely related cultivars. To assess polymorphism, genetic diversity, allele frequencies, and genetic distances, 15 SSR markers were also used to exotic mango types. The identification and exchange of provenances of mango germplasm from different climatic zones of Pakistan for genetic diversification is a significant step in the breeding strategy foundation [21].

In order to guarantee global food security, Salgotra and Chauhan [55] emphasized the significance of protecting and responsibly using plant genetic resources (PGRs). PGRs are a vital resource for increasing agricultural resilience and productivity since they include all of the genetic material found in a crop and its wild relatives. The study underlined that preserving genetic diversity is crucial for sustainable farming and long term food security because of the growing global population and environmental changes that are causing genetic erosion and the extinction of vital resources.

This knowledge of the genetic variation between different forms of mango, including wild relatives, makes it easier for plant breeders to decide on which characteristics will enhance fruit yield, disease resistance and quality. Genetic resources have been effectively utilized in conventional breeding, primarily for the selection of seedlings and the introduction of genes through hybridization to produce new varieties [56, 57]. This is especially the case in relation to Pakistan where mangoes are one of the leading export crops and hence play a role in the economy of the country. Dillon et al. [56] discovered more than 1,000 SSR motifs. Out of them, 25 EST-SSR markers pertaining to fruit characteristics, stress response, and plant development were created and examined in 32 mango cultivars. In order to differentiate between all genotypes, twenty-four markers exhibited polymorphism, yielding 86 alleles (avg. 5.38/locus). By enhancing parentage identification and hybrid breeding techniques, these markers improve the Australian Mango Breeding Program’s current SSR panel.

The variation in the size, shape and color of fruits in the collected germplasm is an indication of the variability and variation in the agro-climatic regions of Pakistan. Such variation in mango fruit structures is relevant as it provides the basis for the selection of distinct features that may prove valuable in improvement of the mango growing practices. For example, the data on the germplasm collected showed significant differences in fruit size among the varieties. Varieties of multifold Sindhri found in Sindh and Chaunsa found in Punjab had larger fruit sizes as compared to varieties found in Khyber Pakhtunkhwa and Baluchistan had comparatively smaller fruit sizes.

These results support the previous findings of Chandio et al. [27], who also identified the differences in fruit size among different varieties of mangoes originating from the various regions of Pakistan. The shape of the mango fruits in the collected germplasm also differentiates which includes an oval shape as seen in Fajri from Sindh and a round shape as seen in Langra from Punjab. It is also essential for marketing and customers’ acceptance of the fruits since the shape of fruits may differ in various markets. In addition, the color of the mango fruits ranged from green to yellow while some of the varieties like Sindhri and Malda having yellow-colored fruits looked more attractive in the market. Apart from the aesthetic value, the variations in the color of fruits also have the effect of signaling the level of ripeness of fruits, as observed by Shahbaz et al. [29]. Besides the fruit size, shape and color other observed morphological characters in the collected germplasm include skin thickness, pulp texture and aroma. These traits are useful in the identification of the appropriate mango varieties for various purposes like fresh fruit usage, processing or export. For instance, some of the varieties with tough skin and firm pulp like Sindhri are better suited for transport and processing to dried mangoes or mango juice. Dillon et al. [56] made this same observation emphasizing the significance of skin thickness and pulp texture when defining mango varieties for specific markets.

In the present study, the SSR marker based genetic diversity study has given detailed information regarding the extent of polymorphism and genetic variation in the mango cultivars. By selecting SSRs, these genomic regions were illustrated to be very efficient in evaluating genetic variability and distinctiveness since all the markers displayed a hundred per cent polymorphism scores across the loci. In detail, the study established a total allelic number of 2–6 for the locus and the PIC that was obtained was 0.25 to 0.78. It is also important to note that the PIC value is an essential marker of informativeness and the bigger the PIC value the better will distinguish genotype. The average shall be less than or equal to the unity; while the average PIC value is 0.694 in this study proving that the SSR markers utilized are suitable in that they can identify allelic variations that are significant for genetic differentiation in mango. These results corroborate previous studies in mango and other fruit species, where SSR markers have also been appreciated for their high information content and for the opportunity they offer to unveil the amount of genetic diversity [15, 26, 56]. Similarly, using 14 SSR markers, Ravishankar et al. [9] examined 387 Indian mango accessions and identified two major genetic subpopulations, “South-West” and “North-East,” with notable divergence (FST = 0.248). The South-West had greater diversity, and AMOVA validated variation both within and between groups. In order to characterize mangos, six SSR loci were found to be universal markers. These variations observed for different SSR loci amplify the fact that a lot of genetic diversity exists in the mango germplasm. Using cluster analysis that classified the cultivars according to their gene similarities, there was a definite separation between some of the clusters, hence implying that the cultivars in question might be of the same genetic stock or have been under similar selective pressures during the domestication and breeding processes. For instance, samples of the cultivars originating from certain geographical locations grouped: this could mean that certain varieties were developed due to regional adaptation or due to breeding. These genetic clusters are therefore important in detecting and conserving the specific genetic diversity within the mango fruit crop of that specific region. Other investigations show similar populations’ similarities in mango and other fruit tree crops where it was applied to breeding approaches and genetic resource conservation [26, 58, 59]. The new allelic patterns described in this study contribute to the genetic resource database of mango germplasm. For example, some alleles identified from this study were not described in the prior studies, meaning that there are newly identified genetic polymorphic forms that have not been described before. These novel alleles could be associated with relevant characteristics including fruit size, flavor or disease resistance hence are potential candidates for future breeding efforts. These new alleles indicate the genetic resources remaining unexplored in the germplasm of mango thus the new cultivar can be developed with improved characteristics. Using SSR markers, Drabo et al. [60] examined 18 mango cultivars in Burkina Faso and discovered 21.49% polymorphism with genetic distances between 0.0002 and 1.09. Cultivars were divided into two major clusters by dendrogram analysis, which also revealed substantial genetic diversity that may be used to improve germplasm for future breeding.

Recent studies using SSR markers demonstrate that both small ~ 9–12 loci [22, 61], and large (~ 34–40 loci [60, 62] SSR panels can effectively resolve genetic diversity and population structure across *Mangifera indica* germplasm at both local and global scales (Table 7).

**Table 7:** Current achievements in assessing mango genetic diversity by SSR markers [22, 60–63]

Study / Year	SSR markers	Germplasm source	Major findings
Yan et al., 2024 [22]	~ 34	Global (231 accessions)	High allelic diversity, clear population structure clusters
Drabo et al., 2024 [60]	~ 10–18	Burkina Faso	Moderate polymorphism, two major genotype groups
Hussein et al., 2023 [61]	10	Saudi cultivars	Good diversity, loci under selection, non-geographic clustering
Tran et al., 2024 [62]	9	Vietnam (Ba Den Mountain)	High polymorphism (~ 94%), three cluster groups
Tang et al., 2023 [63]	40	China (188 accessions)	High diversity, PIC ~ 0.58, clusters matched geographic patterns

These analyses are important to reveal the organization of population mango, widely selected for the formation of plantation and conservation programs mango. The SSR marker data indicated that mango cultivars belong to more than one genetic group but certain cultivars share more similarities and are more related than others. In this research noted that these groupings frequently matched the geographic origin of the cultivars and implied that the local climate and breeding practices influenced the genetic composition of these mango populations. For instance, cultivars originating from the Punjab region like ‘Chaunsa’ and ‘Anwar Ratol’ fruit types had turned out to be closely related to each other and Australian cultivars due to geographical affinity and similar selection pressures. Conversely, cultivars originating from Sindh province, including ‘Sindhri,’ were grouped separately, there implying a probable genetically shift that could be attributed to the differences in the agro-climatology of Sindh and Punjab provinces. This means that mangoes followed a regional clustering pattern, which is supported by other researches carried out to analyze genetic relatedness on mango and other fruit trees [26, 56]. The recognition of these clusters is very important to the process of this mango breeding programs as this way breeders are able to find genetically different parents from different clusters and hence be able to create hybrids with high heterogeneity in the offspring. Further, the clustering relationship can also be useful in determining possible sources of resistance genes to diseases or other stresses usually in certain areas. For example, it is possible that some of the cultivars belonging to some of the clusters may be more resistant to some form of stress, say drought, or disease and such character can be bred into the other cultivars. Similar works in other parts of the world have also acknowledged the cluster analysis, in advising breeding by recognizing subpopulations in a particular crop species [26, 58, 59].

The principal component analysis (PCA) assessment conducted in this study also helps to extend the understanding of the genetic makeup of the mango cultivars since it decreases the dimensions of data and reveals the latent components responsible for most of the variability in the matter. The first two principal components (PCs) explained a considerable level of genetic variation; the first PC, 45% and the second was 25%. This suggests that several genetic elements, which influence mango characteristics, account for the majority of variation when compared to the diverse mango cultivars. Likewise, the PCA outcomes aligned with the data presented in the cluster analysis, where multiple cultivars clustered together were likely to be clumped together in the PCA plot. For example, clusters of Punjab and Sindh cultivars were the same in both analyses; again, PCA supports these findings of cluster analysis that Punjab and Sindh cultivars are genetically distinct. Further, the PCA pointed out some cultivar outliers, which do not belong to any specific group and could contain novel genetic variation that is valuable for breeding. These may be cultivars possessing rare alleles or those that may have been subjected to different selective pressures by either natural or artificial selections as compared to the main groups. The consistency between the PCA and cluster analysis results enhances the validity of the genetic relationships observed in this study. However, the PCA also sheds further light that is not obtained from cluster analysis only, such as the recognition of the exact genetic features (PCs) that explain the differentiation of the cultivars. These insights are valuable for decoding the genetic basis of the mango germplasm and could inform more precise breeding strategies. Similar to the previous studies, the present study also used the PCA complementing the cluster analysis for the genetic diversity where both methods can be so powerful to give a better idea about the genetic structure of the populations under study [64]. Similarly, genetic diversity and pedigree analysis of Chinese mango accessions showed two heterotic groups (P1 and P2) with substantial hybridization, resulting in 80.99% mixed ancestry, according to Liang et al. [5]. The results point to new approaches to managing germplasm and breeding mangoes.

## Conclusions

This research shows the genetic variability and population differentiation in Pakistani mango germplasm, besides establishing the presence of high allelic variation in the population and significant single nucleotide polymorphisms related to desirable fruit traits. The SSR markers used in this study were shown to be useful in evaluating genetic variability in mango, hence the prospects of adopting them in mango breeding programs. The conclusion indicates the significance of synergizing conventional breeding techniques with molecular biologists to generate high-yielding, climate-tolerant mango varieties. Despite the given potential of Pakistan germplasm of mango, line-down difficulties like restricted genetic variation and environmental stress are crucial constraints. Through proper tackling of these challenges through breeding and conservation strategies, the production of mangos in Pakistan and other regions can be sustained.

## Data Availability

No DNA or RNA sequencing has been carried out in the present study. The data generated or analyzed during the current study are available upon reasonable request from the corresponding author (Prof. Shazia Iram).

## Abbreviations

AMOVA: Analysis of molecular variance
DNA: Deoxyribonucleic acid
He: Expected heterozygosity
Ho: Observed heterozygosity
PC: Principal component
PCA: Principal component analysis
PCoA: Principal coordinate analysis
PCR: Polymerase chain reaction
PIC: Polymorphic information content
SSR: Simple sequence repeat
